# Strategic roadmap for FAIR-compliant transcriptomic workflows: from technical challenges to bioinformatics democratization

**DOI:** 10.3389/fbinf.2026.1882808

**Published:** 2026-10-12

**Authors:** Verónica Jiménez Jacinto, Leticia Vega-Alvarado

**Affiliations:** 1 Unidad Universitaria de Secuenciación Masiva y Bioinformática, Instituto de Biotecnología, Universidad Nacional Autónoma de México, Cuernavaca, Morelos, Mexico; 2 Instituto de Ciencias Aplicadas y Tecnología, Universidad Nacional Autónoma de México, Ciudad de México, Mexico

**Keywords:** bioinformatics democratization, differential gene expression, FAIR principles, IDEAMEX, RNA-seq, transcriptomic workflows, transcriptomics

## Abstract

This article draws on the authors’ more than 17 years of experience in transcriptomic research, beginning with the introduction of high-throughput sequencing in Mexico in 2009. Here, we provide a critical review of bioinformatics pipelines for different RNA classes, ranging from small RNAs to long non-coding RNAs. Our analysis encompasses a range of scenarios, from de novo transcriptome assembly for organisms lacking a closely related and adequately annotated reference genome to differential expression analysis based on a well-annotated reference genome. By providing a comprehensive overview of the workflow, from RNA extraction and high-throughput sequencing to quality control and data preprocessing, we offer a practical framework to help researchers select tools that are appropriate for their biological questions and experimental contexts. We further examine advanced strategies for transcriptome assembly, sequence alignment, and transcript abundance quantification. Finally, we discuss differential expression and functional enrichment analyses, emphasizing the importance of standardized, FAIR-compliant workflows and the democratization of transcriptomic analysis in promoting inclusive and reproducible genomic research. We also present IDEAMEX, a web-based platform designed to democratize transcriptomic analysis by reducing technical and systemic barriers and promoting equitable access to robust analytical tools, particularly for researchers in resource-limited settings who may not have advanced programming skills.

## 1 Introduction

The advent of high-throughput RNA sequencing (RNA-seq) has transformed our understanding of gene regulation, cellular function, and disease mechanisms. In Mexico, this paradigm shift began in 2009 with the installation of the country’s first Illumina sequencing system at the University Unit for High-Throughput Sequencing and Bioinformatics (UUSMB) of the UNAM Institute of Biotechnology. Although the adoption of these technologies has contributed to the expansion of bioinformatics research across the country, women remain underrepresented in the field, as they are in other STEM disciplines (according to Mexico’s researcher registry database: https://www.datos.gob.mx/dataset/sistema_nacional_investigadoras_investigadores_snii_s191). In this context, their achievements are particularly noteworthy given their limited access to essential computing resources, the scarcity of targeted mentoring and training opportunities, and institutional inequalities that can hinder their professional development.

Whole-transcriptome analysis, which examines total RNA within a biological sample, has become a cornerstone of contemporary molecular biology because it enables the expression of both coding and non-coding genes to be characterized in a single experiment. This comprehensive approach has enabled a wide range of transformative applications across diverse fields. In agricultural and animal sciences, it has facilitated important advances in veterinary parasitology (Reyes-Guerrero et al., 2023), teleost physiology (Corona-Herrera et al., 2022), and plant-pathogen interactions (Solano-De la Cruz et al., 2019). It is also an important tool in genomic medicine for investigating pathological mechanisms (Beltrán-Hernández et al., 2025), in pharmacology for conducting molecular-level studies across organisms (López-Teros et al., 2025), and in structural biology for examining the functions of small RNAs during transcription (Sánchez-Correa et al., 2022).

However, transcriptome analysis involves extensive data processing and requires careful consideration of the algorithms used at each stage. Based on our experience at the UUSMB, we have observed that every step of the workflow, from initial data processing to final interpretation, can introduce biases that substantially affect biological conclusions (Conesa et al., 2016; Mandelboum et al., 2019; Molania et al., 2023). These technical challenges, together with the computational demands of transcriptomic analyses, can pose significant obstacles for researchers seeking to develop the necessary bioinformatics skills.

The objective of this study is to provide an expert perspective and strategic evaluation of key algorithms used in transcriptomic analysis, drawing on more than 17 years of experience in the field. We examine how the choice of workflow depends on the availability of genomic resources and consider three major scenarios: the use of well-annotated reference genomes, the use of closely related annotated organisms to facilitate transcriptome assembly, and the more challenging task of *de novo* assembly for non-model species. Finally, we evaluate how these strategies should be adapted to different RNA classes, including messenger RNA (mRNA), small RNA (sRNA), and long non-coding RNA (lncRNA).

## 2 Strategic methodological framework for transcriptomic workflows

A robust transcriptomic workflow begins with RNA extraction, followed by sequencing using high-throughput sequencing platforms. These technologies generate raw data in FASTQ format containing sequencing reads derived from the transcriptome. In the following sections, we evaluate the different stages of a contemporary transcriptomic study and highlight the key methodological decisions required to ensure biological validity and reproducibility.

### 2.1 Workflows in RNA-seq

In the field of RNA-seq, the most widely used approaches include mRNA, sRNA, and total RNA sequencing (Conesa et al., 2016; Coussement et al., 2024; Lee et al., 2024). mRNA sequencing has become a widely used approach for analyzing transcriptomes in pathological conditions (Beltrán-Hernández et al., 2025), biological processes (Vichi et al., 2021), and a diverse range of experimental settings. In addition to providing sensitive and accurate measurements of gene expression (Li and Dewey, 2011; Patro et al., 2017), mRNA-seq enables the identification of transcript isoforms, gene fusions, and other known and novel transcriptomic features, as well as allele-specific expression. mRNA-seq based on next-generation sequencing (NGS) provides a comprehensive view of the coding transcriptome without requiring extensive prior knowledge of transcript structure (Kukurba and Montgomery, 2015; Wang et al., 2009). Furthermore, strand-specific mRNA-seq can provide information on transcript orientation, enabling the detection of antisense transcripts, more accurate quantification of overlapping transcripts, and a higher proportion of mappable reads (Levin et al., 2010). Non-coding sRNAs play important roles in gene silencing and post-transcriptional regulation of gene expression (Mahamadou et al., 2026). sRNA sequencing enables the isolation and sequencing of sRNA species, including microRNAs (miRNAs). RNA-seq can facilitate the analysis of thousands of sRNA and miRNA sequences with high sensitivity and a broad dynamic range. Total RNA sequencing provides a comprehensive approach for detecting coding and multiple forms of non-coding RNA. It enables the quantification of gene and transcript abundance and the identification of known and novel transcriptomic features. In particular, total RNA sequencing facilitates the simultaneous analysis of coding RNA and multiple classes of non-coding RNA, including lncRNA, thereby providing a more comprehensive view of the transcriptome (Stark et al., 2019).

### 2.2 Experimental design

If one of the objectives of a transcriptomic study is to perform differential expression analysis, the experimental design should include a baseline or reference condition, such as a wild-type condition, and a comparison condition. The latter may represent an experimental treatment resulting from a specific environmental condition, drug exposure, or disease state, or may involve a mutant or genetic variant, a different tissue, or another biologically relevant comparison. Biological replication is also an essential consideration when designing transcriptomic experiments. A substantial body of literature has demonstrated that increasing the number of biological replicates can improve the reliability and interpretation of experimental findings (Schürch et al., 2016). We recognize that increasing the number of biological replicates can substantially increase experimental costs and may introduce logistical challenges. However, adequate biological replication is essential for accurately estimating biological variability and improving the statistical power of differential expression analyses. A robust experimental design should distinguish biological variability from technical variation to ensure the reliability of transcriptomic findings. The batch effect is a form of technical variation that occurs when biological samples are processed in different batches, on different days, in different laboratories, or by different technicians. This systematic bias has the potential to obscure or misrepresent genuine biological variations in high-throughput experimental settings. This can have a deleterious effect on research by generating false positives—identifying genes as “differentially expressed” when in reality the difference is due to the day they were sequenced. This phenomenon can also result in a diminution of statistical power, owing to the fact that the technical noise tends to exceed the biological signal of interest. The batch effect represents a significant source of technical variation, occurring when samples from the same experimental condition are prepared or sequenced at different times, by different personnel, or under inconsistent environmental conditions (Leek et al., 2010; Coussement et al., 2024). Drawing on more than 17 years of experience at the UUSMB, we have found that RNA extraction is a particularly sensitive stage of the workflow, during which small differences in processing time, temperature, and reagent lots can introduce systematic variation and increase dispersion in the resulting data. To detect such technical artifacts, exploratory analyses, including principal component analysis (PCA) and multidimensional scaling (MDS), can be performed on normalized expression data, ideally using the most variable genes. These analyses can help identify clustering patterns associated with processing batches rather than biological conditions. Once identified, batch effects can be accounted for by including batch as a covariate in statistical frameworks such as DESeq2 or edgeR, thereby improving the ability to detect genuine differentially expressed genes (DEGs).

At the UUSMB, three biological replicates are generally considered an accepted minimum, although they should not necessarily be regarded as the optimal number. However, Schürch et al. (2016) reported that increasing the number of replicates (e.g., 6–12 replicates) can substantially improve the detection of DEGs. Although three replicates have traditionally been used as a minimum for differential expression analysis, this number may be insufficient to adequately capture biological variability or detect small-magnitude expression changes (Schürch et al., 2016). As reported in several studies (Corona-Herrera et al., 2022; López-Teros et al., 2025; Reyes-Guerrero et al., 2023; Sánchez-Correa et al., 2022), satisfactory results can be obtained with at least three biological replicates for detecting genes with expression changes greater than twofold (log_2_FC ≥ 2), particularly when between-replicate variability is low or when relatively homogeneous biological materials, such as cell lines, are used. However, studies involving patient samples or outbred animal models, including fish, generally require more biological replicates because of substantial inter-individual variability. Six or more biological replicates may provide a useful balance between experimental cost and the statistical power needed to detect DEGs, although the optimal number should be determined based on the expected biological variability and effect size (Liu et al., 2014; Schurch et al., 2016). This recommendation applies specifically to biological replication. Technical variation in RNA-seq is relatively low and predictable under well-controlled experimental conditions, and therefore does not introduce significant downstream bias (Marioni et al., 2008). Overall, increasing the number of biological replicates generally increases statistical power and improves the ability to distinguish biological effects from background variation.

In addition to the number of replicates, sequencing depth should be considered when designing an RNA-seq experiment. Several published RNA-seq studies have successfully identified DEGs using approximately 10 million reads per sample (Vichi et al., 2021; Reyes-Guerrero et al., 2023; Corona-Herrera et al., 2022). However, these examples should not be interpreted as establishing a universal sequencing-depth threshold, because the required depth depends on transcriptome complexity, transcript abundance, biological variability, expected effect sizes, and the objectives of the study (Sims et al., 2014). Lower sequencing depths may be sufficient for detecting highly expressed transcripts, whereas greater sequencing depth can improve the detection of lowly expressed genes in complex transcriptomes (Sims et al., 2014). Therefore, sequencing depth should be considered together with biological replication and the specific objectives of the experiment rather than being determined solely by the organism under study. Finally, the RNA preparation strategy should also be considered during experimental design. Depletion or removal of highly abundant ribosomal RNA (rRNA) can increase the proportion of sequencing reads allocated to informative transcripts and thereby influence the effective sequencing depth (Conesa et al., 2016).

### 2.3 mRNA extraction

mRNA extraction is a molecular biology procedure designed to isolate messenger RNA from other cellular components, including DNA, proteins, tRNA, and rRNA. Unlike total RNA, mRNA represents only a small fraction of cellular RNA, typically accounting for approximately 2%–5% of total RNA. A distinguishing feature of most mature eukaryotic mRNAs is the presence of a poly(A) tail at the 3′ end. Therefore, to selectively construct an mRNA-seq library and reduce the sequencing of highly abundant ribosomal RNA, mRNA can be enriched by capturing polyadenylated transcripts with poly (dT) oligonucleotides coupled to magnetic beads. Alternatively, researchers seeking a more comprehensive transcriptomic profile can use ribosomal RNA depletion to retain non-polyadenylated non-coding RNAs (Perez-Perri et al., 2021).

The primary approaches for RNA isolation include silica-based columns, organic extraction reagents such as TRIzol, and magnetic beads. Magnetic bead-based methods are widely used in downstream library preparation and can be employed either for the positive selection of mRNA using oligo (dT) beads or for the negative selection and removal of ribosomal RNA (rRNA), which constitutes approximately 80%–90% of total RNA. The latter approach increases the proportion of sequencing reads derived from transcripts of interest (Hrdlickova et al., 2017; Kukurba and Montgomery, 2015).

For sRNA analysis, silica membranes with appropriately adjusted ethanol concentrations can be used to selectively retain or recover small RNA fractions, typically those shorter than 200 nucleotides (Head et al., 2014). To enrich regulatory sRNAs, such as those approximately 18–30 nt in length, and remove adapter dimers during library preparation, samples have traditionally been subjected to polyacrylamide gel electrophoresis (PAGE), followed by excision of the corresponding band (Baran-Gale et al., 2015; Head et al., 2014). Automated electrophoretic and bead-based size-selection methods are also increasingly available. Importantly, oligo (dT) enrichment is generally unsuitable for most sRNAs because they lack a poly(A) tail. Instead, specialized single-stranded adapters are directly ligated to the RNA molecules during library preparation (Baran-Gale et al., 2015).

For lncRNA analysis, rather than selectively capturing the target transcripts, a negative-selection strategy can be used to remove unwanted RNA species. Specifically, targeted probes that bind to ribosomal RNA (rRNA) can be used to facilitate its enzymatic degradation or physical removal from the sample (Cui et al., 2010). This approach is particularly useful because lncRNAs are structurally diverse and can be either polyadenylated or non-polyadenylated (Bush et al., 2017). Consequently, oligo (dT)-based mRNA enrichment may irreversibly exclude lncRNAs that lack a 3′poly(A) tail. Ribosomal RNA depletion can overcome this limitation by retaining both protein-coding mRNAs and non-polyadenylated lncRNAs, thereby providing a more comprehensive representation of the transcriptome (Cui et al., 2010; Bush et al., 2017).

### 2.4 High-throughput RNA-seq

RNA-seq uses high-throughput sequencing technologies to characterize the transcriptome of a cell or biological sample. Unlike microarrays, RNA-seq does not require prior knowledge of the genes or transcripts to be analyzed. In addition to quantifying gene expression, this approach can enable the detection of novel transcript variants, rare splicing events, and low-abundance transcripts. For *de novo* transcriptome assembly, in which reads are assembled without relying on a reference genome, long-read sequencing technologies such as those offered by Oxford Nanopore Technologies (ONT) and Pacific Biosciences (PacBio) can provide important advantages (Hu et al., 2026). One of the main limitations of long-read sequencing is its relatively high cost compared with short-read sequencing (Amarasinghe, S. L., et al., 2020). Depending on the platform and experimental design, long-read sequencing can cost substantially more than Illumina-based short-read sequencing. A major challenge of *de novo* transcriptome assembly using short reads is the difficulty of accurately reconstructing transcript isoforms and repetitive regions from relatively short sequence fragments (Hölzer and Marz, 2019). Long-read sequencing can help address this limitation by generating reads that span much larger portions of, or in some cases entire, transcripts. PacBio platforms such as Revio and Sequel IIe use highly accurate HiFi reads and therefore represent a suitable option for transcriptome sequencing (Logsdon et al., 2020). PacBio HiFi sequencing can achieve very high single-read accuracy, making it particularly useful for resolving transcript structures and isoforms (Wenger et al., 2019). This approach can simplify transcriptome assembly by providing reads that span multiple exons and thereby reduce the uncertainty associated with reconstructing exon connectivity (Hu et al., 2026). An alternative is ONT sequencing using platforms such as MinION or PromethION. ONT can provide long reads and, depending on the workflow, may offer a more accessible option for some applications, although costs remain higher than those of conventional Illumina sequencing. Additionally, ONT supports direct RNA sequencing, which does not require conversion of RNA into cDNA (Garalde et al., 2018). Direct RNA sequencing can reduce some biases associated with reverse transcription and PCR amplification and may also enable the detection of RNA base modifications (Logsdon et al., 2020).

A conventional approach for transcriptome sequencing is short-read sequencing using Illumina platforms, including the NovaSeq 6000, NextSeq 2000, NextSeq 500, and MiSeq (Stoler and Nekrutenko, 2021). These systems are widely used because of their low sequencing error rates, high throughput, and relatively low cost per base (Stark et al., 2019). However, *de novo* transcriptome assembly from short reads requires sophisticated bioinformatics approaches, including software such as Trinity, to reconstruct transcripts from overlapping sequence fragments. This process can produce chimeric sequences or incomplete transcript reconstructions. However, the availability of a well-annotated reference genome can substantially streamline transcript reconstruction and downstream analysis.

For differential expression analysis using a reference genome, Illumina short-read sequencing is often a cost-effective option for studies involving large numbers of samples and biological replicates. First, it is generally more economical than PacBio or ONT sequencing when many samples must be processed, which is particularly important for achieving adequate statistical power in differential expression studies (Conesa et al., 2016). Second, when a suitable reference genome is available, long reads are not necessarily required to reconstruct genes. Short reads, typically ranging from approximately 50–300 bp depending on the sequencing platform and experimental design, can be aligned to the reference genome and used to quantify transcript or gene abundance. Third, Illumina platforms can generate large numbers of reads per sample at relatively low cost, increasing the likelihood of detecting low-abundance transcripts and providing a broad dynamic range for expression analysis (Stark et al., 2019). Single-end reads of approximately 75–100 bp may be sufficient when the primary objective is gene-expression quantification, although the optimal read configuration depends on the experimental design and transcriptome complexity. This consideration is particularly relevant to sRNA sequencing. Paired-end sequencing is generally preferable when transcript structure, isoform reconstruction, or alternative splicing events are of interest because reads from both ends provide additional information for resolving transcript structures (Stark et al., 2019). Even when a reference genome is available, ONT or other long-read technologies may be advantageous for organisms with highly repetitive or structurally complex genomic regions, including some plant and mammalian genomes.

### 2.5 Quality control (QC) and data cleaning

Quality control (QC) and data cleaning are essential components of transcriptomic analysis, particularly for RNA-seq, because errors or technical artifacts introduced during these stages can affect downstream results (Paton et al., 2024). Data cleaning is a multistep process designed to remove technical artifacts, unwanted sequences, and low-quality reads to ensure that subsequent analyses, including differential expression and functional enrichment, are based on reliable data.

The process generally begins with a comprehensive assessment of raw sequencing quality using specialized tools such as FastQC (https://www.bioinformatics.babraham.ac.uk/projects/fastqc/). FastQC can identify adapter contamination, potential sequence contaminants, nucleotide composition biases, and other quality-related issues in sequencing reads.

MultiQC (Ewels et al., 2016) is another useful tool for assessing and summarizing sequencing quality across multiple samples. Following this preliminary assessment, read preprocessing can be performed as needed. Depending on the identified quality issues, preprocessing may include adapter removal, filtering of reads based on average or per-base quality, and trimming of bases from the 5′or 3′ends of reads.

Several tools are available for read preprocessing. Cutadapt (Martin, 2011), for example, can detect and remove adapter sequences, primers, poly(A) tails, and other unwanted sequences from high-throughput sequencing reads. Illumina has incorporated related adapter-trimming approaches into its sRNA sequencing workflows. Another widely used option is fastp (Chen et al., 2018), which integrates adapter trimming, quality assessment, and read filtering into a single workflow. Its implementation in C++ enables rapid processing of large sequencing datasets. Trimmomatic (Bolger et al., 2014) is another commonly used tool for adapter trimming and removal of low-quality bases and reads. This Java-based program has been widely adopted for both RNA-seq and DNA sequencing data. AdapterRemoval (Schubert et al., 2016) provides additional functionality for identifying and removing adapter sequences and for merging overlapping paired-end reads.

For long-read sequencing, NanoPlot can be used to assess read quality, followed by adapter removal and read trimming with tools such as Chopper. These tools are part of the NanoPack2 suite (DeCoster and Rademakers, 2023). For Oxford Nanopore long-read data, Porechop_ABI specializes in adapter detection and removal (Bonenfant et al., 2022). This tool is particularly useful for identifying and removing chimeric or intermediate adapter sequences, thereby improving the integrity of long-read datasets.

### 2.6 *De novo* transcript assembly

When an adequately annotated reference genome is unavailable, *de novo* transcriptome assembly may be required. As discussed in Section 2.4, several sequencing strategies can be used for this purpose. However, no single transcript assembly method can be considered optimal across all datasets and quality metrics. Given the wide range of available software tools (Raghavan et al., 2022), the choice of assembler should instead be guided by factors such as sequencing technology, read length, read accuracy, transcriptome complexity, and the specific objectives of the study. For Illumina short-read RNA-seq data, de Bruijn graph-based approaches are widely used for reconstructing transcripts. Trinity and rnaSPAdes are among the established tools for this purpose.

Trinity uses a three-stage assembly process. First, Inchworm assembles reads by identifying paths through a k-mer graph using a greedy approach, generating a set of linear contigs. Second, Chrysalis groups related contigs and constructs individual de Bruijn graphs for each group. Third, Butterfly processes these graphs by removing spurious edges and simplifying linear paths, followed by reconciliation with the reads and read pairs to reconstruct transcript sequences representing alternative splice forms and paralogous transcripts (Grabherr et al., 2011).

Similarly, rnaSPAdes uses a multistep approach based on a condensed de Bruijn graph. The process includes graph construction and removal of erroneous or chimeric edges, mapping of read pairs to the assembly graph, repeat resolution and scaffolding using the exSPAnder algorithm, and extraction of final transcript and isoform sequences by evaluating paths through the graph (Bushmanova et al., 2019). Combining Trinity and rnaSPAdes may provide complementary information and can be considered when evaluating *de novo* assemblies.

For sequencing performed using PacBio or ONT platforms, RNA-Bloom2 (Nip et al., 2023) is a useful option for *de novo* transcriptome assembly. Recent benchmarking studies have reported strong performance of RNA-Bloom2 on ONT cDNA, ONT direct RNA, and PacBio datasets, considering factors such as assembly accuracy, computational efficiency, and overall performance. The software can process large sequencing datasets and reconstruct transcripts while resolving reads into distinct isoforms. Another option is isONform (Petri and Sahlin, 2023), which is designed for transcriptome assembly from full-length isoform reads generated by Oxford Nanopore or PacBio sequencing. It has demonstrated strong performance in reconstructing alternative transcript isoforms. RATTLE (De la Rubia et al., 2022) is another *de novo* approach designed for long-read data, particularly Oxford Nanopore direct RNA and cDNA sequencing. It performs transcript correction and reconstruction without requiring a reference genome. rnaSPAdes can also be considered as an alternative, although it was originally developed primarily for short-read data. Its applicability to long-read datasets should therefore be evaluated according to the specific sequencing technology and dataset characteristics rather than assumed to be equivalent to its performance with short reads.

When a high-quality reference genome is available, StringTie2 (Kovaka et al., 2019) is a widely used tool for transcript assembly and quantification. For long-read data, StringTie2 can reconstruct transcript structures efficiently and can identify novel isoforms even in the absence of comprehensive reference annotation. More importantly, this software is specifically optimized to manage the elevated error rates characteristic of PacBio and Oxford Nanopore data.

#### 2.6.1 Evaluation of *de novo* transcript assembly

Several computational tools are available for evaluating the quality of *de novo* transcript assemblies. DETONATE (Li et al., 2014), which stands for DE novo TranscriptOme rNa-seq Assembly with or without the Truth Evaluation, consists of two component packages, RSEM-EVAL and REF-EVAL. RSEM-EVAL is a reference-free evaluation method based on a probabilistic model that uses only the assembly and the RNA-seq reads used to generate it. REF-EVAL is a toolkit of reference-based metrics that includes contig-, nucleotide-, and pair-level precision, recall, and F1 scores, as well as a k-mer compression score and several measures comparing k-mer distributions between the assembly and the reference. CATS (Comprehensive Assessment of Transcript Sequences) (Bodulić and Vlahoviček, 2026) also provides both reference-free (CATS-rf) and reference-based (CATS-rb) pipelines for transcriptome quality assessment. CATS-rf maps RNA-seq reads to assembled transcripts and calculates four interpretable scoring components that capture common assembly errors. CATS-rb evaluates transcriptome completeness by aligning assembled transcripts to a reference genome and supports both annotation-free and annotation-based scoring. TransRate (Smith et al., 2016) is an open-source tool for evaluating *de novo* transcriptome assemblies without requiring a reference genome. By analyzing the assembly and mapping reads back to the assembled transcripts, TransRate can identify potential problems such as chimeric transcripts and sequence errors and can be used to compare assemblies and filter low-quality contigs. rnaQUAST (Bushmanova et al., 2016) is another comprehensive tool for evaluating *de novo* transcriptome assemblies. It calculates metrics such as transcript length, N50, complete and partial BUSCO recovery, and GeneMarkS-T statistics. The tool can evaluate assemblies using a reference genome and gene annotations in GTF/GFF format.

The choice of evaluation tool should be guided by the specific objectives of the analysis and the available data because the capabilities of these tools overlap. CATS (Bodulić and Vlahoviček, 2026) is a useful option when both reference-free (CATS-rf) and reference-based (CATS-rb) assessments are desired. When a high-quality reference genome and corresponding GTF/GFF annotation are available, rnaQUAST provides a comprehensive set of assembly-quality metrics. For non-model organisms for which a reference genome is unavailable, TransRate can be used to assess assembly quality based on read support. DETONATE is particularly useful for probabilistic evaluation and comparison of assemblies generated using different parameter settings.

Evaluating assembly quality is only one component of transcriptome reconstruction; the resulting assembly may also require post-processing to reduce redundancy and remove poorly supported transcripts. CD-HIT-EST, part of the CD-HIT package (Li and Godzik, 2006), is a bioinformatics tool designed to cluster nucleotide sequences, including DNA and RNA, according to a specified sequence-similarity threshold. It can be used to reduce sequence redundancy and generate non-redundant sequence databases and is particularly useful for relatively similar or redundant transcript sequences, including assembled transcriptomes and expressed sequence tags (ESTs). Corset (Davidson and Oshlack, 2014) is a command-line tool that groups contigs from *de novo* transcriptome assemblies into gene-level clusters based on shared read-mapping patterns, thereby facilitating downstream differential gene expression (DGE) analysis. This approach helps account for transcript redundancy and can group alternatively spliced transcripts and paralogous sequences before expression quantification.

#### 2.6.2 Annotation of *de novo* transcripts

Following *de novo* assembly and the removal of redundant or low-quality transcripts, the next step is to annotate the reconstructed transcripts. Trinotate is a functional annotation suite designed for *de novo* transcriptomes, particularly those generated using Trinity. It integrates the results of multiple bioinformatics tools into a unified SQLite database, providing functional information for reconstructed transcripts and their predicted proteins. Trinotate serves as a central framework for processing transcript sequences through several functional annotation and prediction pipelines:

- Sequence prediction: TransDecoder (Hass et al., 2013) is used to predict long open reading frames (ORFs) and translate them into predicted protein sequences.
- Homology searches with BLAST (Altschul et al., 1990): BLASTX is used to search transcript sequences, whereas BLASTP is used to search the predicted protein sequences generated by TransDecoder against protein databases such as Swiss-Prot or UniProt to identify homologous proteins.
- Domain identification: HMMER (Eddy, 1998) is used in conjunction with the Pfam database to identify conserved protein domains.
- Feature prediction: SignalP (Teufel et al., 2022) is used to predict signal peptides, and TMHMM (Krogh et al., 2001) is used to identify transmembrane regions.
- Functional annotation: Additional functional information can be assigned using resources such as Pfam (Mistry et al., 2021) for protein families and functional domains, Gene Ontology (GO) terms (geneontology.org) for functional categorization and downstream enrichment analyses, and EggNOG (Huerta-Cepas et al., 2017) and KOG classifications (Tatusov et al., 2003) for broader orthologous-group assignments.

### 2.7 Alignment

Following *de novo* transcriptome assembly or the selection of an appropriate reference genome, the next step is to align the sequencing reads to the reference sequence. When a reference genome is available, splice-aware aligners such as HISAT2 and STAR can be used. When no reference genome is available, reads can instead be aligned to the *de novo* transcriptome assembly using tools such as BWA or Bowtie2, depending on the analytical objective. The resulting alignments can then be used for transcript or gene abundance estimation. More than 100 software tools are available for sequence alignment, making this a fundamental step in many genomic and transcriptomic analyses. Selecting an appropriate aligner from the wide range of available options can therefore be challenging (Mushi et al., 2021). Here, we focus on tools that are widely used and particularly relevant to transcriptomic workflows. These aligners are recognized for their speed, accuracy, memory requirements, and ability to identify splice junctions and other transcriptomic features (Coxe et al., 2024; Mushi et al., 2021).

Hierarchical Indexing for Spliced Alignment of Transcripts 2 (HISAT2) is a splice-aware aligner designed for RNA-seq data and is the successor to TopHat2. It is characterized by high processing speed and relatively low memory requirements, making it suitable for aligning RNA-seq reads to reference genomes. HISAT2 can also identify known and novel splice sites, an important feature for transcriptome analysis. The program uses a hierarchical indexing strategy based on the Burrows-Wheeler transform and the Ferragina-Mannzini (FM) index to efficiently align sequencing reads to the reference genome (Kim et al., 2019). Its indexing strategy can also incorporate information about known variants, such as single-nucleotide variants and small insertions and deletions, when appropriate. Since its introduction as the successor to TopHat2, HISAT2 has been widely adopted because of its combination of speed, accuracy, and relatively low memory usage.

STAR (Spliced Transcripts Alignment to a Reference) is another widely used splice-aware aligner for RNA-seq data and is known for its high speed and sensitivity. The STAR alignment algorithm (Dobin et al., 2013) consists of a seed-searching stage followed by clustering, stitching, and scoring of the candidate alignments. The algorithm identifies splice junctions by mapping seed sequences and extending them across candidate junctions. An important advantage of STAR is its ability to identify splice junctions without requiring a dedicated database of known junctions. STAR uses a suffix-array-based indexing strategy to facilitate rapid mapping of sequencing reads to the reference genome. The algorithm can also identify mismatches and insertions or deletions through its read-extension procedure, thereby improving alignment sensitivity when sequencing errors or genomic variation are present.

The Burrows-Wheeler Aligner (BWA) is a suite of algorithms designed for mapping sequencing reads to reference sequences. Although BWA is primarily used for genomic DNA sequencing, it can also be applied to transcriptomic data (i.e., short reads). BWA-MEME (Jung and Han, 2022), the successor to BWA-MEM (Li, 2013), has been reported to improve alignment speed and accuracy relative to BWA-MEM. However, BWA-MEME is not a splice-aware aligner and therefore does not specifically identify splice junctions or reconstruct transcript isoforms. Consequently, it is best suited for applications involving direct alignment of reads to *de novo* transcript assemblies when splice-aware genome alignment is not required. Bowtie2 combines the Burrows-Wheeler transform and FM-indexing framework with a flexible alignment strategy to provide fast and sensitive short-read alignment (Langmead and Salzberg, 2012). Like BWA, Bowtie2 can be used to align reads directly to *de novo* transcriptome assemblies. It is also incorporated into several transcriptome analysis workflows, including Trinity, and can therefore be useful for assessing read support for assembled transcripts. For long-read alignment, Minimap2 is one of the most widely used aligners. It is designed to efficiently map long sequencing reads to reference genomes or transcriptome assemblies while maintaining relatively low computational requirements (Li, 2018). Its speed, memory efficiency, and accuracy have made it a commonly used choice for long-read data generated by platforms such as Oxford Nanopore and PacBio (Li, 2018).

### 2.8 Transcript counting

A fundamental step prior to differential expression analysis is the construction of an abundance matrix (or count matrix) representing expression levels for the genomic features of interest, such as genes or transcripts. Two main strategies are commonly used for this purpose: alignment-based methods, which rely on reads aligned to a reference genome or *de novo* transcript assembly, and alignment-free or lightweight mapping approaches. No single tool is optimal for all transcript quantification scenarios. In general, tools that provide greater computational efficiency may make different trade-offs in accuracy or sensitivity, whereas methods that perform more detailed alignment or probabilistic modeling may require greater computational resources. Several tools are widely used for quantifying short-read data generated by Illumina platforms, including Salmon (Patro et al., 2017), Kallisto (Bray et al., 2016), RSEM (Li and Dewey, 2011), StringTie (Pertea et al., 2016), HTSeq (Anders et al., 2015), and featureCounts (Lia et al., 2014). For long-read data generated by Nanopore or PacBio platforms, tools such as Bambu, IsoQuant, and Oarfish can be used for transcript and isoform quantification.

Among the most widely used transcript quantification tools, Salmon is primarily recognized as a transcript-level quantifier. It uses lightweight quasi-mapping and selective alignment to quantify reads against a transcriptome reference rather than requiring conventional alignment to the entire genome. This strategy enables rapid quantification while accounting for factors such as fragment length, sequence-specific bias, and positional bias. Salmon consists of an indexing and mapping framework followed by online and offline inference procedures that estimate expression levels and model parameters (Patro et al., 2017). Salmon can also be used with long-read data through appropriate workflow settings, including the--ont option for Oxford Nanopore or PacBio reads. However, because Salmon was originally developed primarily for short-read data, its suitability for long-read quantification should be evaluated according to the sequencing platform and experimental design.

RSEM is another widely used transcript quantification tool and is incorporated into the Trinity workflow. Because it can quantify transcripts against a *de novo* transcriptome assembly, it is particularly useful when a reference genome is unavailable. RSEM estimates transcript and gene abundance using a probabilistic model and reports several commonly used measures, including TPM (transcripts per million). TPM accounts for transcript length and sequencing depth, and the sum of TPM values within a sample is 1 × 10^6^. Unlike RPKM or FPKM, TPM normalization first accounts for transcript length and then normalizes for sequencing depth, facilitating comparisons of relative transcript abundance across samples. RSEM also generates expected counts, which are fractional rather than integer-valued counts representing the expected number of fragments assigned to a gene or isoform by the probabilistic model. These estimates can be used in downstream differential expression analyses, although the appropriate input should be selected according to the requirements of the statistical framework and the specific RSEM output being used. RSEM can also report FPKM or RPKM values, which normalize read or fragment counts according to transcript length and library size. These measures are retained in some workflows for compatibility with legacy analyses, although TPM is generally preferred for reporting relative transcript abundance. RSEM has also contributed to the cost-effective evaluation of sequencing depth and experimental design for RNA-seq quantification (Li and Dewey, 2011).

StringTie is a transcript assembly and quantification tool that estimates the abundance of genes and transcript isoforms (splicing variants) in individual biological samples. It reports commonly used measures such as FPKM and TPM. In addition to short-read data generated by platforms such as Illumina, StringTie can process long-read data from PacBio and Oxford Nanopore platforms and can support analyses that integrate short- and long-read sequencing data.

HTSeq is a widely used toolkit for processing and quantifying RNA-seq data from previously generated alignments, typically produced by aligners such as STAR or HISAT2. HTSeq-count generates raw read counts for individual genes or genomic features and provides a summary of reads that could not be assigned to features. These categories include: no_feature: reads aligned to genomic regions that do not contain annotated genes or exons; ambiguous: reads aligned to regions in which two or more distinct genes overlap, preventing unambiguous assignment to a single gene; too_low_aQual: reads excluded because their mapping quality (MAPQ) falls below the user-defined minimum threshold; not_aligned: reads that were marked as unaligned by the alignment software; alignment_not_unique: reads with multiple reported alignments to different genomic locations. These reads are typically excluded to avoid double counting. featureCounts is another widely used read-counting tool. It generates a count matrix containing the number of reads assigned to each genomic feature and a summary file reporting read-assignment statistics (Liao et al., 2014).

For long-read data generated by Nanopore or PacBio platforms, the selection of a quantification tool depends on factors such as the availability of a reference genome, the sequencing technology, and whether the analysis involves a model or non-model organism. Bambu and IsoQuant are particularly useful for long-read transcript quantification due to their optimal balance between accuracy and sensitivity. Bambu is an R package designed for long-read transcriptome analysis, particularly with Oxford Nanopore data. It can identify novel isoforms and assign reads to transcript models for abundance estimation. Its statistical framework also helps distinguish supported novel transcripts from potential false positives, which is important when analyzing long-read data (Chen et al., 2023). Bambu is therefore well suited to analyses requiring gene- and isoform-level quantification. IsoQuant is designed for accurate and sensitive transcript and isoform quantification from long-read sequencing data. It can process both Oxford Nanopore and PacBio HiFi reads and is particularly useful for characterizing transcript structures and alternative splicing. Its ability to reconstruct and quantify transcript isoforms makes it suitable for detailed analyses of transcriptome structure. Oarfish is a relatively recent tool designed for rapid transcript quantification using probabilistic models. It has demonstrated efficient performance with PacBio Kinnex data and can process large datasets rapidly. Oarfish may therefore be particularly useful for large-scale datasets in which computational efficiency is an important consideration (Jousheghani et al., 2025).

Transcript quantification can generally be performed at either the gene or transcript level. Gene-level quantification involves aggregating reads or fragments assigned to the transcripts or exonic regions of a gene, regardless of their specific splicing patterns. Tools such as HTSeq and featureCounts assign reads to annotated genomic features, most commonly exons, and are widely used to generate gene-level counts for differential gene expression analysis. Transcript-level quantification, in contrast, estimates the abundance of individual transcript isoforms produced by a given gene. Tools such as Salmon and Kallisto use maximum likelihood estimation to estimate transcript abundance while accounting for reads that are compatible with multiple isoforms. Transcript-level quantification is particularly useful for investigating alternative splicing and changes in isoform usage. RSEM, Bambu, and IsoQuant can provide both gene-level and transcript-level abundance estimates, depending on the analysis and output configuration.

### 2.9 Differential expression analysis

The objective of differential expression analysis is to identify genes or transcripts whose abundance differs significantly between two or more experimental conditions. This analysis is a critical component of transcriptomic studies because it can provide insights into biological mechanisms associated with diseases, treatment responses, environmental adaptation, and other biological processes (Beltrán-Hernández et al., 2025; Hernandez-Eligio et al., 2020). Once an abundance or raw-count matrix has been generated, exploratory analysis should be performed to assess sample quality, identify potential outliers, and evaluate the consistency of biological replicates. These preliminary analyses should be completed before statistical testing for differential expression.

Differential expression analysis generally identifies genes or transcripts whose abundance is significantly higher or lower under one experimental condition than under a reference or control condition. The resulting sets of upregulated and downregulated genes can then be subjected to functional enrichment analysis to identify biological processes, molecular functions, pathways, or other functional categories associated with the observed transcriptional changes. The overall workflow is illustrated in Figure 1.

**FIGURE 1:**
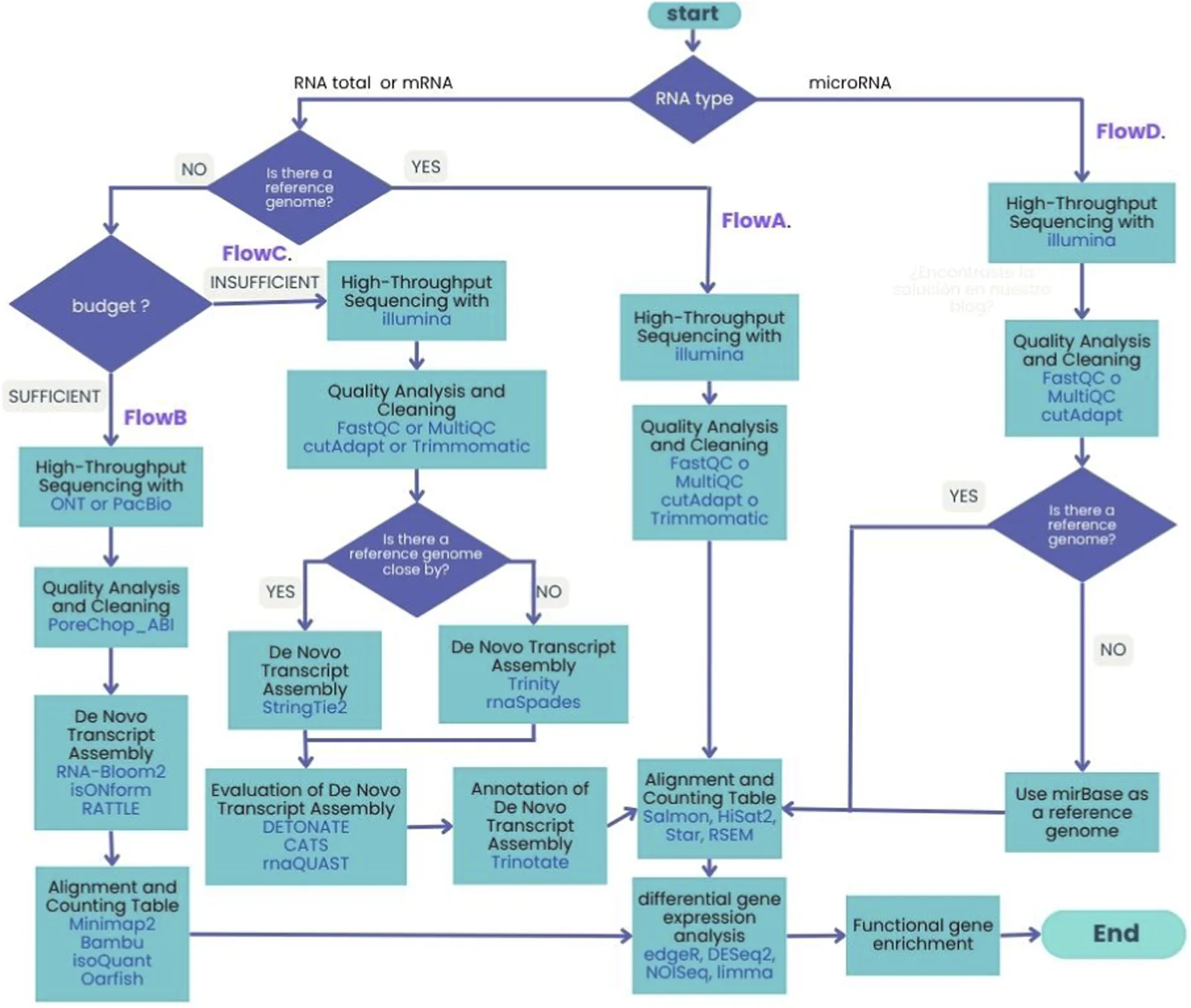
Detailed overview of the transcriptomic workflow. The process begins with classification of the RNA to be analyzed. For mRNA or total RNA, including lncRNAs, the workflow proceeds to the left side of the diagram. If a well-annotated reference genome is available, Workflow A (Flow A) should be followed. This workflow uses paired-end sequencing on Illumina platforms (HiSeq 2000, NextSeq 500, or MiSeq), which can provide a cost-effective sequencing strategy. If no reference genome is available, the choice of workflow depends on the availability of a closely related reference genome, the complexity of the organism’s genome, and the available budget. When no closely related reference genome is available, or when the available reference genome is unsuitable because the organism has a large or highly complex genome, as is the case for many plants and mammals, Workflow B (Flow B) can be used when sufficient funding is available. This workflow involves sequencing with Oxford Nanopore or PacBio technologies. Alternatively, when no reference genome is available and the organism has a relatively small and less complex genome, or when financial resources are limited, Workflow C (Flow C) can be followed. This workflow uses paired-end sequencing on Illumina platforms. When the focus is on sRNA, Workflow D (Flow D) should be followed, using single-read sequencing on Illumina platforms.

#### 2.9.1 Exploratory data analysis

Exploratory data analysis is an important step in assessing the overall quality of an RNA-seq experiment before differential expression testing. Its objectives include identifying potential batch effects, evaluating consistency among biological replicates, and detecting outlier samples (Tarazona et al., 2015). Exploratory analyses are performed using the previously generated abundance matrix or raw counts. Depending on the downstream analysis, count data are typically normalized and transformed before visualization. Common approaches include logarithmic transformation, variance-stabilizing transformation (VST), and regularized logarithmic transformation (rlog) (Love et al., 2014).

PCA and MDS are among the most commonly used approaches for visualizing relationships among samples and assessing separation between experimental conditions. As noted above, these analyses are generally performed using normalized and transformed expression values, often focusing on the most variable genes rather than the complete set of genes. Box plots can be used to assess the distribution of expression values across samples, whereas heatmaps with hierarchical clustering can help determine whether samples from the same experimental condition exhibit similar gene expression profiles.

An important but sometimes overlooked preprocessing step in RNA-seq DGE analysis is the filtering of lowly expressed genes before statistical testing. Genes with very low counts provide limited information for differential expression analysis and may contribute disproportionately to estimates of dispersion and model uncertainty (Giwa et al., 2026). Therefore, it is useful to examine counts per million (CPM) across samples before differential expression analysis and to use this information to establish appropriate filtering criteria. Packages such as edgeR, NOISeq, and DESeq2 provide built-in approaches for filtering low-count genes. Appropriate filtering can reduce the influence of uninformative features and improve the efficiency and statistical power of downstream analyses. Overall, exploratory analysis facilitates the identification of technical problems and the assessment of data quality before differential expression testing.

#### 2.9.2 Methods for differential expression analysis

Numerous statistical approaches have been developed for differential expression analysis (Costa-Silva et al., 2023). Among the most widely used methods, DESeq2 and edgeR use negative binomial models to account for biological variability and overdispersion in RNA-seq count data. The limma framework, when combined with the voom transformation, instead uses linear models after modeling the mean-variance relationship of log-transformed expression values. These methods are widely established in RNA-seq analysis because they provide robust statistical frameworks for modeling biological variability and can accommodate a range of experimental designs, including studies with relatively small numbers of biological replicates (Love et al., 2014; Robinson et al., 2010). Recent reviews have likewise identified DESeq2, edgeR, and limma among the most commonly used approaches for RNA-seq differential expression analysis (Costa-Silva et al., 2023).

DESeq2 and edgeR can be broadly classified as parametric approaches because they specify probability distributions for count data and estimate model parameters, including dispersion, from the observed data. These methods are particularly appropriate for experiments with biological replication because replicate data are essential for estimating biological variability. In contrast, non-parametric approaches such as NOISeq (Tarazona et al., 2011) do not rely on a predefined parametric distribution for the observed counts. Instead, NOISeq estimates the empirical distribution of technical and biological noise from the data or through simulations and uses this information to assess the probability of differential expression. These approaches may be useful under certain experimental conditions, particularly when distributional assumptions are difficult to satisfy. Nevertheless, the absence of biological replicates substantially limits the reliability of statistical inference, regardless of the differential expression method used.

##### 2.9.2.1 DESeq2

DESeq2 uses statistical models based on the negative binomial distribution to analyze RNA-seq count data. This framework accounts for biological variability and overdispersion, which are characteristic of transcriptomic count data. The method incorporates normalization based on size factors, estimates gene-specific dispersion, and subsequently performs statistical testing to identify DEGs. P-values are adjusted for multiple testing using the false discovery rate (FDR), providing a controlled framework for identifying statistically significant differential expression (Love et al., 2014). DESeq2 also allows for pre-filtering of low-count genes during result extraction, which can improve statistical power by excluding genes with insufficient information from multiple-testing correction. Low-count genes can also be pre-filtered before model fitting to reduce computational requirements. A simple filtering threshold, such as a minimum count of 10 across a specified number of samples, can be used as a practical starting point, although filtering criteria should be adapted to the experimental design and library sizes (Love et al., 2014).

##### 2.9.2.2 edgeR

edgeR is another widely used differential expression package based on negative binomial models for RNA-seq count data. A key feature of edgeR is its empirical Bayes approach to dispersion estimation, which allows information to be shared across genes and can improve variance estimates, particularly in experiments with relatively few biological replicates. edgeR also incorporates normalization methods that account for differences in RNA composition between libraries and provides statistical tests suitable for a wide range of experimental designs. edgeR retains genes that have a sufficiently high count in a minimum number of samples. Before statistical testing, edgeR can filter out genes with insufficient expression using the filterByExpr () function (Robinson et al., 2010; McCarthy et al., 2012; Anders et al., 2013). This function determines an appropriate filtering threshold based on library sizes and the experimental design, thereby retaining genes with sufficient expression in an appropriate number of samples. This filtering step reduces the influence of low-information genes on downstream analyses.

##### 2.9.2.3 NOISeq

NOISeq is a non-parametric method for differential expression analysis of RNA-seq data. Unlike approaches based on predefined count distributions, NOISeq does not assume a specific parametric distribution for the observed data. Instead, it estimates the noise distribution empirically from the data or through simulations. Differential expression is assessed by comparing expression levels between conditions while considering both the magnitude of the observed changes and the variability in the data (Tarazona et al., 2011). NOISeq provides several filtering options, including CPM, Wilcoxon, and Proportion, which can be implemented using the filtered. counts () function.

##### 2.9.2.4 Limma

Originally developed for microarray data, the limma (Linear Models for Microarray Data) package has been successfully adapted for RNA-seq analysis through the voom transformation. Unlike DESeq2 and edgeR, which directly model count data using negative binomial distributions, limma-voom uses linear models on transformed expression values. The voom method converts counts into log-counts per million (log-CPM), estimates the mean-variance relationship, and assigns precision weights to individual observations. These weights allow the linear modeling framework to account for the mean-dependent variance characteristic of RNA-seq data. Limma-voom is computationally efficient and can accommodate complex experimental designs, making it particularly useful for studies involving larger sample sizes or multifactorial experimental designs (Ritchie et al., 2015).

Differential expression analysis ultimately produces a set of genes or transcripts whose abundance differs significantly between experimental conditions. However, identifying statistically significant changes is only the first step in interpreting transcriptomic data. The next step is to determine the biological significance of these changes through functional enrichment and pathway analyses.

### 2.10 Biological interpretation

Following the identification of DEGs, the next step is to interpret their biological significance through functional enrichment analysis. This approach assesses whether specific biological processes, molecular functions, cellular components, or metabolic pathways are significantly overrepresented among the genes of interest. Functional enrichment analysis is particularly important in transcriptomic studies because these experiments often identify large numbers of DEGs, making it difficult to interpret their individual biological significance. Rather than analyzing each gene independently, enrichment methods identify coordinated patterns associated with specific cellular functions, biological processes, or molecular pathways.

The most commonly used annotation databases for this purpose include Gene Ontology (GO), the Kyoto Encyclopedia of Genes and Genomes (KEGG), and Clusters of Orthologous Groups (COG). GO classifies gene products into three main categories: biological processes, molecular functions, and cellular components. KEGG organizes genes and gene products into metabolic pathways, signaling pathways, and other functional networks. COG, an NCBI database, classifies proteins from complete genomes, particularly those of bacteria and archaea, into functional groups based on orthology, referring to genes that originated from a common ancestral gene and diverged following speciation.

Functional enrichment analysis can be broadly classified into three categories according to the underlying algorithms and the manner in which input data are processed:

1. Singular Enrichment Analysis (SEA)/Over-Representation Analysis (ORA): This approach evaluates whether specific functional terms are statistically overrepresented in a predefined list of genes relative to a reference background set. The gene list is typically defined using statistical thresholds, such as a specified *p*-value or adjusted *p*-value (García-Moreno et al., 2022; Huang et al., 2009b; Wijesooriya et al., 2022; Xu et al., 2024). Statistical significance is commonly assessed using the hypergeometric distribution or Fisher’s exact test (Huang et al., 2009b; Hosack et al., 2003; Draghici et al., 2003).
2. Gene Set Enrichment Analysis (GSEA)/Functional Class Scoring (FCS): This approach analyzes the transcriptomic dataset as a whole without requiring a predefined significance threshold for individual genes. Genes are ranked according to a biological or statistical criterion, and the method evaluates whether predefined gene sets are enriched toward the top or bottom of the ranked list. This approach can therefore detect coordinated changes involving genes with relatively small individual expression changes (García-Moreno et al., 2022; Huang et al., 2009b; Subramanian et al., 2005; Xie et al., 2011).
3. Modular Enrichment Analysis (MEA): This approach incorporates relationships among functional annotations or biological networks to identify groups of correlated terms. By considering interactions among annotations, MEA facilitates the identification of biological modules rather than treating functional terms as independent entities (García-Moreno et al., 2022; Huang et al., 2009b).

Several computational tools are currently available for functional enrichment analysis. Among the most widely used are DAVID (Dennis et al., 2003; Huang et al., 2009a) and the GSEA (Gene Set Enrichment Analysis) software package (Subramanian et al., 2005; Liberzon et al., 2011; Wijesooriya et al., 2022). Other widely used tools include g:Profiler (Reimand et al., 2007; Kolberg et al., 2023), PANTHER (Mi et al., 2013; Mi et al., 2019), KOBAS (Wu et al., 2006; Xie et al., 2011; Bu et al., 2021), and WebGestalt (Zhang et al., 2005; Elizarraras et al., 2024). The topGO package (Alexa and Rahnenführer, 2009; 2010) is another widely used tool that accounts for the hierarchical structure of GO terms during enrichment analysis, thereby reducing the potential effects of dependencies among related terms. Finally, GREAT (McLean et al., 2010) is widely used for functional interpretation of genomic regions by associating noncoding genomic regions with nearby or functionally related genes and biological annotations (Chicco and Jurman, 2022).

## 3 FAIR principles

A key consideration in modern transcriptomic workflows is compliance with the FAIR principles: Findable, Accessible, Interoperable, and Reusable (Wilkinson et al., 2016). This consideration is particularly important because RNA sequencing data are complex and costly to generate and can be affected by substantial technical variability. Ensuring that sequencing data, associated metadata, and analytical workflows adhere to FAIR principles promotes scientific reproducibility and maximizes the long-term value of research data. Data traceability requires the use of unique and persistent identifiers throughout the analytical workflow for input files, transcripts, genes, and datasets. Examples include GEO or BioProject accession numbers and stable gene or transcript identifiers rather than gene names alone. Such identifiers facilitate the retrieval of raw sequencing data, including FASTQ files, and enable researchers to trace the origin and processing history of the data. Comprehensive metadata are also essential for facilitating data discovery through indexed searches and should include detailed descriptions of experimental conditions, sample characteristics, sequencing procedures, and analytical workflows. Accessibility requires the deposition of sequencing data and associated files in public repositories that provide long-term storage and standardized access. Examples include the NCBI Sequence Read Archive (SRA), the European Nucleotide Archive (ENA), and the DNA Data Bank of Japan (DDBJ). Where appropriate, processed data, such as gene- and transcript-level quantification matrices, should also be made available through repositories or supplementary resources using standardized and accessible formats. Providing these data reduces technical barriers to data retrieval and facilitates independent analysis and verification. Interoperability depends on the use of standardized data formats and controlled vocabularies. Formats such as BAM, GTF/GFF3, and CSV facilitate compatibility with a broad range of bioinformatics software and programming environments, including R, Python, and command-line tools. The use of standardized ontologies and controlled vocabularies, such as GO, is also important for consistently describing phenotypes, cell types, biological processes, cellular components, and molecular functions. These practices reduce ambiguity in nomenclature and facilitate the integration of information across biological databases and analytical platforms. Reusability requires comprehensive documentation of data provenance, analytical code, input and output files, software versions, parameter settings, reference genomes, and applicable usage licenses. For example, software versions such as Bambu v3.0 and IsoQuant v3.3 should be explicitly reported, together with the parameter values used for each analysis. Reference genome assemblies and annotation versions should also be specified so that other researchers can reproduce the analytical workflow and integrate the resulting datasets with other studies. The FAIR principles therefore have implications throughout the entire transcriptomic workflow, from validation of input files to the identification and validation of novel transcripts, generation of gene- and transcript-level expression matrices, and publication of processed results in standardized formats such as GTF/GFF3 or CSV. These practices facilitate independent verification and reuse of the data by other laboratories and research groups. Reproducibility can be further strengthened by preserving the computational environment in which the analysis was performed. Containerization and environment-management tools, such as Docker and Conda, can help preserve software dependencies and versions, thereby improving the reproducibility of quantitative results obtained from transcript quantification, isoform identification, and functional enrichment analyses.

## 4 Conclusion

Transcriptomic analysis workflows can follow multiple pathways depending on biological, computational, and economic considerations. Key biological factors include the availability of a reference genome or a sufficiently well-annotated genome from a phylogenetically related organism, as well as the type of RNA being analyzed, including total RNA, lncRNA, and sRNA. Computational capacity and access to specialized infrastructure also influence the selection of appropriate analytical strategies. The careful selection of experimental and bioinformatics strategies, including the number of biological replicates, quality control procedures, and methods for transcriptome assembly, read alignment, and differential expression analysis, is essential for obtaining robust and biologically reliable results that can support downstream analyses. The most appropriate workflow depends on the biological characteristics of the organism under investigation, the availability and quality of reference genomes and annotations, computational resources, and the budget allocated to the study.

Despite the increasing availability of high-quality reference genomes, *de novo* transcriptome assembly using tools such as RNA-Bloom2 and isONform remains valuable in modern transcriptomics. This approach is particularly useful for studying non-model species and can facilitate the detection of genomic rearrangements and chimeric transcripts, as well as the identification of novel isoforms and lncRNAs while reducing dependence on existing annotations. For long-read sequencing and reference-free transcriptome analysis, Oxford Nanopore and PacBio technologies provide powerful options. For short-read sequencing in the absence of a reference genome and under budget constraints, Trinity or rnaSPAdes, as well as hybrid approaches, remain useful alternatives.

Alignment methods such as STAR and HISAT2 account for exon–intron structures and alternative splicing, thereby supporting the accurate construction of abundance matrices for downstream differential expression analysis. Appropriate normalization is likewise essential and should be tailored to the characteristics and distribution of high-throughput sequencing data to minimize bias in subsequent analyses.

In many experimental settings, differential expression analysis methods such as DESeq2 and edgeR can provide reliable results when sufficient biological replication is available. Although methods such as NOISeq can be useful when the number of biological replicates is limited, reduced replication generally decreases statistical power and can increase uncertainty in differential expression estimates. Therefore, conclusions drawn from datasets with limited replication should be interpreted cautiously.

Functional enrichment analysis of genes or genomic regions that differ between experimental and control conditions facilitates the interpretation of the biological context underlying RNA-seq data. By linking changes in gene expression to biological processes, molecular functions, cellular components, and pathways, these analyses provide a broader understanding of the biological mechanisms associated with transcriptional changes.

The continued expansion of bioinformatics has also led to the development of new tools and analytical pipelines that are increasingly accessible to users without extensive expertise in large-scale data analysis, high-performance computing, or command-line interface (CLI)-based applications. Such developments, when implemented in accordance with FAIR principles, can reduce technical barriers to transcriptomic analysis. As a contribution to this effort, we developed IDEAMEX (Jimenez et al., 2019), a web-based platform designed to facilitate transcriptomic analysis from abundance matrices. The platform supports exploratory data analysis and differential expression analysis using four methods: DESeq2, edgeR, limma, and NOISeq. Integration of the results generated by these methods can provide complementary evidence for identifying robust differential expression patterns and selecting datasets for subsequent functional enrichment analysis. The value of IDEAMEX therefore extends beyond facilitating data processing to improving access to bioinformatics resources for communities with limited computational resources.

The democratization of bioinformatics extends beyond the availability of analytical algorithms and requires efforts to reduce technical, infrastructural, and social barriers to participation. The complexity of transcriptomic workflows and the need for specialized computational infrastructure represent critical obstacles, disproportionately affecting women in science and researchers in resource-constrained settings. In this context, IDEAMEX is presented not only as a technical resource but also as a means of improving accessibility to transcriptomic analysis for users without advanced programming expertise. Combined with the adoption of FAIR principles, such resources can contribute to more open, reproducible, and inclusive access to genomic knowledge, regardless of available computational infrastructure or financial resources.
