# Supplementary material for: Strategic roadmap for FAIR-compliant transcriptomic workflows: from technical challenges to bioinformatics democratization
Source: Front Bioinform. 2026 Oct 12;6:1882808. doi: 10.3389/fbinf.2026.1882808 (PMC13696915; doi:10.3389/fbinf.2026.1882808)
Supplement: Supplementary file 1 [file DataSheet1.pdf]

Appendix A. Example workflows for differential expression analysis of bulk RNA-seq count data

The following examples illustrate representative workflows for the differential expression analysis stage of a bulk RNA-seq experiment using DESeq2 and edgeR. The workflows begin with a raw gene-level count matrix and corresponding sample metadata and therefore do not cover upstream steps such as sequencing quality control, adapter or quality trimming, read alignment or pseudoalignment, transcript or gene quantification, or generation of the count matrix. These upstream procedures are outside the scope of the present examples. The code is intended to illustrate the main steps involved in differential expression analysis, including low-count filtering, normalization, statistical modeling, differential expression testing, and multiple-testing correction.

A.1 Input data and sample information

The workflows assume that gene-level raw count data have already been generated from the RNA-seq experiment. The input consists of a matrix of raw integer counts, with genes as rows and biological samples as columns, together with sample metadata describing the experimental conditions.

```
# Count matrix
counts <- read.csv("counts.csv", row.names = 1)
# Sample metadata
coldata <- read.csv("metadata.csv",row.names = 1)


# Ensure identical sample order
coldata <- coldata[colnames(counts), ]


# Define experimental condition
coldata$condition <- factor(coldata$condition,levels = c("control", "treated"))
```

A.2 DESeq2 workflow

DESeq2 models count data using a negative binomial distribution and estimates size factors for normalization and dispersion parameters for modeling biological variability.

```
library(DESeq2)
# Create DESeq2 dataset
dds <- DESeqDataSetFromMatrix(countData = counts,colData = coldata,design = ~ condition)
# Pre-filter low-count genes
smallestGroupSize <- min(table(coldata$condition))
keep <- rowSums(counts(dds) >= 10) >= smallestGroupSize
dds <- dds[keep, ]


# Differential expression analysis
dds <- DESeq(dds)
# Extract results
res <- results(dds,contrast = c("condition", "treated", "control"))
# Order by adjusted p-value
res <- res[order(res$padj), ]
# Export results
write.csv( as.data.frame(res),"DESeq2_results.csv")
```

This workflow illustrates the main steps of DESeq2 analysis, including optional pre-filtering of low-count genes, model fitting using a negative binomial framework, extraction of differential expression results, and adjustment of p-values for multiple testing.

A.3 edgeR

edgeR also models RNA-seq count data using a negative binomial distribution. Its workflow includes library-size normalization, dispersion estimation, and statistical testing within a generalized linear model framework.

```r
library(edgeR)
# Create DGEList object
y <- DGEList(counts = counts,group = coldata$condition)
# Filter lowly expressed genes
keep <- filterByExpr( y,group = coldata$condition)
y <- y[keep, , keep.lib.sizes = FALSE]


# TMM normalization
y <- calcNormFactors(y)


# Create design matrix
design <- model.matrix(~ condition,data = coldata)


# Estimate dispersion
y <- estimateDisp( y,design)
# Fit negative binomial GLM
fit <- glmQLFit(y,design)


# Test treated versus control
qlf <- glmQLFTest(fit,coef = "conditiontreated")
# Extract results
res <- topTags(qlf,n = Inf)$table
# Export results
write.csv( res, "edgeR_results.csv")
```
This workflow illustrates the main steps of edgeR analysis, including expression filtering with filterByExpr(), TMM normalization, dispersion estimation, negative binomial generalized linear modeling, and differential expression testing.
